# Buprenorphine maintenance program with contracted work/education and low tolerance for non-prescribed drug use: a cohort study of outcome for women and men after seven years

**DOI:** 10.1186/s12888-015-0415-z

**Published:** 2015-03-24

**Authors:** Leif Öhlin, Mats Fridell, Anna Nyhlén

**Affiliations:** 1 Department of Psychiatry, Lund University Hospital, SE 221 85 Lund, Sweden; 2 Department of Psychology, Lund University, SE 221 01 Lund, Sweden

**Keywords:** Outcome, Gender, Co-morbidity, Buprenorphine, Maintenance program, Treatment contract, Low tolerance for illicit drug use, Personality disorder, Crime

## Abstract

**Background:**

A seven-year follow-up of heroin dependent patients treated in a buprenorphine-maintenance program combining contracted work/education and low tolerance for non-prescribed drug use. Gender-specific differences in outcome were analysed.

**Methods:**

A consecutively admitted cohort of 135 men and 35 women, with eight years of heroin abuse/dependence on average was admitted to enhanced buprenorphine maintenance treatment. Standardized interviews, diagnostic assessments of psychiatric disorders and psychosocial conditions were conducted at admission and at follow-ups. Outcome associated with gender was reported for abstinence, retention, psychiatric symptoms, employment and criminal convictions.

**Results:**

148 patients started treatment. After seven years, 94/148 patients (64%) were retained in the program, employed and abstinent from drugs and alcohol. Women had more continuous abstinence, retention and employment than men (76% versus 60%). After one year patients with a high-risk consumption of alcohol were no longer heavy consumers of alcohol and remained so throughout the study (p < .001). All women regained custody of their children.

At admission, more women than men had been admitted for psychiatric disorders (70%/44%) and to compulsory care for substance abuse (30%/18%). Initial gender differences of psychiatric co-morbidity decreased and were no longer significant after one year.

More men than women had been imprisoned (62% versus 27%) or in non-institutional care (80% versus 49%). Criminal convictions were reduced from 1751 convictions at admission to 742 (58%) after seven years.

Eight patients in the entire cohort died over the 7 years (0.7% per year). One patient died in the completers group while still in the program (0.1% per year).

**Conclusions:**

After seven years, two thirds of the patients in the program were abstinent and employed. Convictions ceased in the completers group. One patient died in the completers group.

Women had superior long-term outcome compared to men: more continuous abstinence, employment and fewer convictions. Women also lived with their children to a higher extent than men.

The positive outcome highlights the importance of maintaining high structure in combining pharmacological treatment with a focus on employment and psychological treatment and low tolerance for non-prescribed drug use.

## Background

### History and epidemiology of drug abuse in Sweden

In the past decade, the abuse of opiates in Sweden decreased, whereas the illegal use of buprenorphine, tramadol hydrochloride, alcohol and other drugs increased in the European Union [1]. In Sweden, the number of *heavy users* defined as individuals “who have injected drugs during the past 12 months or used drugs daily/almost daily for the last four weeks” has remained constant about 30,000 of whom more than 10,000 use opiates as the dominant drug [1,2].

### Long-term outcome of substance abuse and dependence

Three studies with a 20 to 33-year follow-up of defined cohorts have reported on the long-term course of heroin-dependent patients. The proportions of drug-free and deceased persons were as follows: 25% versus 23% [3], 41% versus 18% [4], 5% versus 40% [5], respectively. A five-year follow-up study of a consecutively admitted sample of 125 patients with co-morbid psychiatric disorders and drug dependence showed that 39% had been abstinent for two years or longer and 17% percent had five years of continuous abstinence since discharge [6]. Women showed a stable improvement in 55% compared to males (30%). At a fifteen-year follow-up of the same sample, stable abstinence dropped from 39% to 31%, whereas those continuously abstinent for five years remained so at fifteen years. The incidence of premature death was 1% - 1.2% per year over the 15 years [7].

### Maintenance treatment of heroin addiction

The low proportion of abstinent opiate abusers in long-term follow-ups as well as the high mortality in dropouts from the drug-free therapeutic communities emphasized the need for pharmacological interventions. Since the 1960s, methadone maintenance treatment has been used in Sweden. Due to the official negative attitude to methadone maintenance in Sweden before 2005, a maximum of 800 patients were allowed to enter the maintenance programs [8]. In Sweden buprenorphine was introduced as an alternative to methadone in 1999. The present study reports one of the first attempts to implement buprenorphine treatment in a Swedish clinical cohort.

Pharmacologically, buprenorphine is a partial opioid agonist which eliminates abstinence without producing euphoria. The effectiveness of buprenorphine is comparable with that of methadone, but the side effects are fewer and the intoxication and dependence potential is lower than for methadone or other opioids when administrated sublingually [9-13]. Today maintenance treatment has gained wide acceptance in Sweden.

International studies emphasize the importance of high structure for compliance, retention and positive outcome of maintenance programs [9,14-19]. According to the Swedish guidelines, methadone or buprenorphine therapy should be prescribed in combination with psychological and social interventions [19,20].

The risk of premature death is a major problem in opiate abuse/dependence. A 37-year follow-up of a cohort of substance dependent patients treated at a detoxification ward in the 1970’s (n = 561), reported a premature death rate in opiate dependent patients of 36.4%. The average age of drug related death was 39.9 years [21]. The increased risk of premature death was identical in a population study in Stockholm made by an independent research team [22]. Methadone maintenance treatment and more recently buprenorphine maintenance have been regarded as the most life-saving treatment interventions for opiate dependence. The rate of premature death has constantly been higher among patients who dropped out or were discharged from maintenance treatment than among patients in treatment. In a Swedish study, the death rate among dropouts was as high as in untreated patients [23]. A Norwegian study of a national cohort of 3221 patients in maintenance treatment reported a death rate of 4% during treatment [24].

In the Norwegian cohort study, males with a stable retention of two years or more had a 36% reduction of convictions versus 57% for females over the treatment period. Women had 42% fewer convictions during treatment than men [24]. A majority of the patients (78%) had never been convicted prior to treatment entry compared to 18% of the males and 24% of the females in a Swedish methadone program [23].

A major problem in maintenance treatment is the use of non-prescribed drugs and illicit substances which increase the risk of relapse and overdose [25,26]. More recently the abuse potential of methadone and buprenorphine has attracted growing attention [27]. During the past ten years premature death increased within the maintenance programs in Stockholm, Sweden [28]. This reflects, according to Romelsjö et al. [8], a more permissive attitude among treatment staff to illicit drug use and non-prescribed medications in the maintenance programs. It also indicates that the quality of care and the clinical management of relapses and side-misuse were sometimes compromised. Even when applying evidence-based methods like methadone or buprenorphine maintenance with defined regulations of care [9,20] the adherence to rules and contingencies may differ from one treatment unit to another.

The debate has often focused on the increased risk of premature death if patients were discharged [23,25]. The present program focused on strategies to help the patient avoid relapses and substance use in the first place. Consequently, social and medical support but not buprenorphine were provided by the team during the 3 months’ suspension period following discharge. The aim of this procedure was to reduce the risk of premature death and non-fatal overdoses.

### Psychiatric disorders and criminal activity

In epidemiological as well as in clinical cohort studies, substance dependent patients display more prevalent and more serious psychiatric disorders than alcohol-dependent individuals do [6,29,30]. Substance (narcotic) dependent patients display a high incidence of personality disorders (50-80%) often overlapping with depression and anxiety disorders (30%-60%) and ADHD (20%) but with a lower proportion (8-9%) of chronic psychoses [31-37]. The personality disordered patients in the “dramatic cluster” in particular, display problems with impulsivity, emotional instability and conflicts in close relations [31,33,35,38].

The most prevalent co-morbid personality disorder is antisocial PD (minimum 25%) when DSM-IV-TR is used [6,31,32,34,35]. Antisocial personality disorder is more prevalent among men than among women who more often have a histrionic or borderline personality disorder [6,34,35]. In a cohort of women in compulsory care for substance dependence, however, the prevalence of antisocial personality disorder was the same as for men in compulsory care [35]. Antisocial personality disorder is associated with early problem behaviour and conduct disorder. About half of the teenagers diagnosed with conduct disorder in childhood developed an antisocial personality disorder in adulthood [38]. Typical life style features of antisocial personality disorder are impulsivity and criminal behaviour and low compliance with treatment interventions [31-33].

### Gender

The proportion of men versus women with “heavy daily use” in the general population in Sweden has been stable over the past twenty years with 25% women in the population of substance users and 33% in clinical samples [39,40]. Many substance-dependent women complain about gender aspects of treatment being neglected or not taken properly care of in the drug treatment programs [41,42].

Social disruptions in childhood, poor home conditions, mental illness as well as abuse of alcohol and drugs were common in the homes of both male and female substance users [41,43]. Significant gender differences were reported in a long-term follow-up of children of fathers with alcohol problems where girls responded to poor childhood conditions with more “neurotic” and somatic symptoms while, in contrast, boys more often developed alcohol dependence and antisocial personality [44-46].

#### Aims

The first aim of the study was to evaluate the seven-year outcome of a buprenorphine-assisted treatment program with contracted work/education combined with low tolerance for non-prescribed drug use. The second aim was to analyse the influence of gender and psychiatric disorders on outcome of substance use, social situation and criminal activity.

## Methods

The design was a prospective clinical study with observation design in a cohort of patients consecutively admitted to the program from August 1st 2004 to December 30th 2011 (n = 170). All 170 patients were diagnosed by ICD 10 and DSM-IV-TR/SCID II in addition to assessment with a standardized battery of biological and psychological measures at intake and at regular follow-up intervals. There is a risk that patients do not dare to provide full information to the staff about drug use and criminal activity facing the risk of discharge. The general approach in this study was consequently to use objective data as far as possible.

Supervised urine samples were collected at admission and three times a week during the first eight weeks. After 9 weeks the number of checks was reduced to twice a week and after three months to once a week. In addition, random extra supervised urine samples were collected during the whole treatment period. All positive urine samples were sent for independent control to the Department of Clinical Chemistry at the Scania University Hospital. Gas chromatography mass spectrometry (GC-MS) was used for the toxicological analyses. When non-prescribed drug use was suspected, at least two independent positive urine samples were required before a decision on discharge was taken. The reason was to avoid risk of discharge based on non-validated information.

The outcome criteria were compared for gender on the following dimensions: (1) abstinence from drug-/alcohol use, (2) retention in the program, (3) employment, (4) psychosocial conditions and (5) criminal activity. Abstinence was followed by toxicological analyses of alcohol/drugs and benzodiazepines in urine and blood over the entire study [47,48]. Retention in the program refers to days and months of continuous care. Psychological status was followed by several tests and social conditions including employment were documented continuously. Compliance/adherence to treatment was defined as use of prescribed medications only. The indicator of criminal activity was number of convictions.

Clinical interviews were conducted at base line and after one year [49,50]. Follow-up interviews were conducted at four years and at seven years. Personality disorders (SCID-II), personality profiles (SSP) and psychiatric diagnoses were obtained after four weeks [51-53]. Three tests: SCL-90, SOC and AUDIT were collected at 1, 3, 6, 9 and 12 months during the first year [54-60].

The number of convictions were followed from five years prior to admission and two years after the end of the study (2013) by register data (n = 170) obtained from the National Council for Crime Prevention (BRÅ). Causes of death and psychiatric diagnoses from hospital admissions were followed by register data from the National board of Health and Welfare. Patient data from the project were linked to register data by using the patients’ unique national identification ID with the Swedish Central Person Register. Autopsy protocols were obtained in addition to death certificates from forensic clinics in Sweden.

### Treatment contract

The maintenance treatment was multimodal, designed to meet the complex problems of heroin users with or without co-morbid psychiatric disorders. The treatment introduction started with a net-work meeting at the rehabilitation unit with officials in the patient’s home municipality who cooperated with the detoxification and rehabilitation unit and the local employment agency.

The patients were informed about the treatment structure and asked to take part in the research study. The assessment was co-ordinated by the medical social worker (LÖ) and data were also collected by the physician and by the nurses. All prospective patients were informed about the five basic elements: pharmacological treatment, prohibition of side misuse, access to drug-free accommodations, achieving structured employment and continuous psychosocial treatment session each week during the first year. All conditions were mandatory but the patients’ voluntary participation and responsibility were stressed. When all five prerequisites were met, a preparation period of 4–6 weeks preceded the treatment start. If the patient accepted the treatment conditions, a written consent containing the Code of Ethics of the Ethics Committee at Lund University was presented (Reg. 847/2004). A contract was signed which also waived the confidentiality to other network participants.

#### Inclusion criteria

a) a minimum age of 20 years, b) at least 1 year of documented heroin addiction, c) not expelled from another maintenance program during the past three months, d) contact with social services, e) a current drug-free living environment f) an employment contract on starting treatment and g) agreement to the rule that any use of narcotics or non-prescription drugs is prohibited and causes discharge.

#### Exclusion criteria

a) psychosis, b) impending detention in prison or compulsory care, c) not able to speak or understand Swedish.

### Preparations before starting treatment

Prior to the entry, benzodiazepines and methadone were tapered. The setting of buprenorphine treatment was closely monitored to minimize discomfort. Abstinence treatment was completed within the first four weeks of the program with supervised urine samples collected three times a week. During abstinence treatment the participants’ overall health was screened, and blood samples were collected to detect blood-borne infections as hepatitis, HIV and alcohol problems by alcohol markers: CDT, B-PEth [48].

A unique feature of the present maintenance program was the addition of a treatment regimen with a contract in which the patients agreed not to use any non-prescribed drugs while also working or studying. Use of drugs caused discharge. The long-term objective was to support a radical lifestyle change.

### Psychosocial interventions

Parallel to employment/study, the patient had a scheduled treatment session once a week for at least 1 year. The psychosocial treatment included manual-based cognitive-behaviour therapy, psychodynamic or family-oriented counselling [9]. The goal was to modify the patient’s drug use behaviour and to prevent passivity. Continued psychosocial contact after the first year was offered as part of the treatment. Regular network meetings with all officials involved formed another integral part of the treatment as well as continuous reports on attendance or absences from jobs or studies.

### Assessments

For all patients who were enrolled to the program, a semi-structured interview similar to the Addiction Severity Index (ASI) [49] and the Swedish DOK-system (Documentation systems in addiction treatment) [50] was conducted. The interview questionnaire was structured around the same seven well-defined problem areas as in ASI [49]. Interviews were made at intake, after one month, after 12 months, four years and seven years follow-up.

Swedish Universities Scales of Personality (SSP) with 13 subscales, an improved version of the earlier Karolinska Scales of Personality [51] and the Structured Clinical Interview for personality disorders according to DSM-IV-TR - SCID II comprising 120 yes/no items were obtained at index [52,53].

#### Three tests

The Symptoms Checklist (SCL-90), Sense of coherence (SOC) and the Alcohol Use Disorders Identification Test (AUDIT) were assessed during the first year: at 1, 3, 6, 9 and 12 months. The Symptoms Checklist (SCL-90) is a measure of psychological symptoms consisting of nine symptom scales with three summary scales [54,55]. The total score on all scales; the Global Severity Index (GSI) is calculated. All raw scores in the tests were computed into linear T scores corrected for age and sex.

Sense of Coherence (SOC) [56-58] measures the experience of meaning/context based on the total score and three subscales. The cut-off value for “normal function” is a raw-score of 132 points for men and 123 for women. Values over 125 indicate a moderate to high sense of coherence [57]. The Alcohol Use Disorders Identification Test (AUDIT) for alcohol screening [59,60] contains 10 items, with a maximum score of 40. Recommended cut-off score, Rp ≥ 6 for women and Rp ≥ 8, for men indicates a hazardous, harmful use or dependence. Results on tests, clinical scales and diagnostic assessments were reported back to the patient as part of the treatment with the intent to foster the therapeutic alliance.

### Statistics

Statistical comparisons were based on those 148 patients who stayed in the program for a minimum of 30 days and started buprenorphine maintenance treatment. For comparisons of proportions, a χ^2^-test was used. In comparing interval data or data with higher metrical properties analysis of variance and a t-test were employed. Bivariate regression analysis was used to investigate possible differences in the study group. The raw score in the tests was converted to linear T-scores corrected for age and sex differences. The T-scale has 50 as mean with 10 as the standard deviation.

## Results

### Patient characteristics

All consecutively enrolled patients (n = 170, 135 men and 35 women) had a documented heroin dependence of eight years on average (SD = 5, range 2–15 years) and fulfilled the requirements of admission to maintenance treatment prescribed by The National Board of Health and Welfare [19,20]. Age was on average 34.2 years (range: 22–62, Sd = 8.8) for males and 32.2 years (range: 21–52, SD = 8.3) for females. Of the 170 patients, twenty-two (13%) discontinued treatment within 30 days during the 2004–2011 period, subsequently categorized as non-completers. The remaining sample (n = 148) was followed from August 2004 to December 2011 with a continuous retention of 43 months in average and a maximum of 88 months.

After 2010, when new local programs were started, 16 (11%) patients were voluntarily transferred to a buprenorphine program geographically closer to their own home town, where the same treatment principles were applied. These 16 individuals were included in the outcome calculations due to the homogeneity of the interventions at the two sites.

Ninety-nine patients (67%) had injected drugs for 13 years or more. The male patients had a history of a significantly higher proportion of non-lethal overdoses than females: 78% vs. 56%, (p < .05). In addition to heroin addiction, cannabis abuse was more common in men than in women (p < .05). No significant differences were found for gender with regard to the frequency of other substances.

Of patients with alcohol abuse/dependence according to AUDIT (T > 70), 33 individuals also had values of B-PEth associated with high consumption of alcohol (Table 1). Of all the patients 65% tested positive for hepatitis C, but only a few for hepatitis B at baseline. Most patients in the programme had been vaccinated for hepatitis A and B at admission. Only one male tested HIV-positive which reflected the low prevalence of HIV in substance abusers in Sweden.

**Table 1:** **Drug test at admission for men and women from 2004 to 2011**

	All (N = 148)		Men (N = 115)		Women (N = 33)	
Benzodiazepines	74	50.0	57	49.6	17	51.5
Cannabis	56	37.8	48	41.7	8	24.2
Amphetamine	22	14.9	18	15.7	4	12.1
Dextropropoxyphene	5	3.4	3	2.6	2	6.1
Cocaine	1	0.7			1	3.0
Methadone	10	6.8	7	6.1	3	9.1
Opiates	73	49.3	59	51.3	14	42.4
Buprenorphine	68	45.9	54	47.0	14	42.4
Alcohol abuse/depend.	33	22.2	25	21.7	8	24.2

Absolute numbers and percent.

The majority of the patients in this study had suffered a disruptive home environment, broken families and abuse in the family with parents having little or no education. Parental substance use in childhood was reported by 91 (62%) of the patients. Maternal substance abuse was more common among men than among women (78% versus 64%). Women reported more severe psychosocial problems than men during childhood (70% versus 60%).

Table 2 shows that 24 patients (16.2%) were born abroad, and 57 (39%) of their parents were first generation immigrants compared to 6.9% foreign immigrants in the Swedish population [61].

**Table 2:** **Background characteristics of men and women, 2004 – 2011**

	*All (N = 148)*	*Men (N = 115)*	*Women (N = 33)*
**Age**	33.76 (21–62)	34.29 (22–62)	32.23 (20–52)
**Birth country**			
Sweden	124 83.8	95 82.6	29 87.9
Scandinavia	7 4.7	5 4.3	2 6.1
Eastern Europe	13 8.8	11 9.6	2 6.1
Other countries	4 2.7	4 2.7	
**Social conditions**			
Single	109 73.6	86 74.8	23 69.7
Married/cohabiting	39 26.4	29 25.2	10 30.3
Children	72 48.6	54 47.0	18 54.5
Contact - children, 0 days	14 19.7	14 26.4	
Last 30 days	23 32.4	14 26.4	9 50.0**
**Employment**			
Work	33 22.3	26 22.6	7 21.2
Studies	1.7		1 3.0
Sick leave	5 3.4	4 3.5	1 3.0
Subsidized wage compens.	14 9.5	11 9.6	3 9.1
Unemployed	95 64.2	74 64.3	21 63.6
**Residence**			
Private leases	63 42.6	44 38.3	19 57.6*
Parents apartment	23 15.5	22 19.1*	1 3.0
Support apartment	36 24.3	29 25.2	7 21.2
Intrinsic/cohabiting	26 17.6	20 17.4	6 18.2
**Education**			
Elementary school	145 98.0	114 99.1	31 93.9
Not completed high school	52 35.1	36 31.3	6 48.5
High school	50 33.8	39 33.9	11 33.3
Vocational training	42 28.4	35 30.4	7 21.2
Municipal adult education	9 6.1	3 2.6	6 18.2***
University	6 4.1	4 3.5	2 6.1
**Age at onset drug - range**			
Tobacco	11.94 5-27	11,91 15-27	12,03 7-19
Alcohol	12.84 5-19	12,83 5-19	13,33 8-19
Drugs	14.61 9-37	14,66 9-37	14,45 11-20
**Treatment experience**			
Outpatient	111 75.0	84 73.0	27 81.8
Treatment voluntary	102 68.9	78 67.8	24 72.7
Compulsory care (LVM)	31 20.9	21 18.3	10 30.3*
**HIV and hepatitis**			
HIV	1.7	1 .9	
Hepatitis C-positive	95 64.2	74 64.3	21 63.6
Hepatit B-positive	2 1.4	2 1.7	
Tested no hepatitis	53 35.8	41 35.7	12 36.4
Vaccinations	96 64.9	73 63.5	23 69.7
**Psychiatric treatment**			
Psychiatric treatment	74 50.0	51 44.3	23 69.7**
Any medication	93 62.8	65 56.5	28 84.8**
Any antidepressant	83 56.1	59 51.3	24 72.7*
Any anxiolytic	36 24.3	23 20.0	13 39.4*
Any neuroleptic	15 10.1	9 7.8	6 18.2
**Criminality**			
Sentenced to prison	80 54.1	71 61.7**	9 27.3
Probation previously	108 73.0	92 80.0***	16 48.5
Current probation	60 40.5	49 42.6	11 33.3
Months in prison (M, SD)	14.4 (24.2)	17.3 (26.0)**	4.2 (11.6)

* p < 0.05;** p < 0.01;*** p < 0.001.

Absolute number, range and percent.

### Social conditions and employment at admission

The majority of the patients lived alone (74%). Of the men 29 (25.2%) and of the women 10 (30.3%) were married or cohabiting and 55% had children. All the women had regular contact with their children but only few lived with their children when enrolled to the program.

At the admission, 64% of the patients in the cohort (n = 170), had no previous work experience as compared with 6.9% in the Swedish population at large (Statistics Sweden 2012 [61]). Twenty-two percent of the patients had been employed periodically and about 10% had previous subsidized wage compensation. There were no gender differences. At the 7-year follow-up, all completers were employed (94/148) in contrast to the dropouts 54/148 and non-completers (22/170), who were all unemployed. At treatment start men more often than women depended on their parents for accommodations, were homeless or lived in apartments provided for by the social services (p < .05).

### Psychiatric symptoms and personality disorders

Proportionally more women than men had a treatment contact in psychiatry at baseline (p < .01). Women also used more antidepressants (p < .01), anxiolytics (p < .05) and neuroleptics (p < .05) than men. At the intake interview, 63 patients (42%) reported suicidal ideation during the past twelve months, men to the same extent as women.

None of the patients in the sample had a schizophrenia spectrum disorder. Bipolar disorder was diagnosed in 16/148 patients at baseline: 5 women (15.2%) versus 11 men (9.6%). At baseline 125/148 (84%) also had at least one personality disorder (DSM-IV-TR, SCID-II) while 18 men (15.7%) and 5 women (15.2%) did not meet criteria for any personality disorder diagnosis. Of those who had a PD diagnosis, most had more than one.

Antisocial personality disorder was the most frequent type in both males and females, 73% versus 64%, respectively (SCID items). Females were more often diagnosed with a borderline, schizotypal and obsessive personality disorder. Narcissistic personality disorder was the only PD with a significantly higher proportion of men to women (59% versus 39%), (p < .05), (see Table 3).

**Table 3:** **Personality disorders according to DSM-IV-TR- SCID II**

Personality disorders	All (N = 148)	Men (N = 115)	Women (N = 33)
DSM-IV-TR	Number Percent	Number Percent	Number Percent
No personality disorder	23 15.5	18 15.7	5 15.2
*Cluster A*			
Paranoid	31 20.9	25 21.7	6 18.2
Schizotypal	55 37.2	41 35.7	14 42.4
Schizoid	8 5.4	7 6.1	1 3.0
*Cluster B*			
Antisocial	105 70.9	84 73.0	21 63.6
Borderline	63 42.6	46 40.0	17 51.5
Histrionic	4 2,.7	3 2.6	1 3.0
Narcissistic	81 54.7	68 59.1*	13 39,4
*Cluster C*			
Avoidant	46 31.1	35 30.4	11 33.3
Dependent	12 8.1	9 7.8	3 9.1
Obsessive-Compulsive	43 29.1	30 26.1	13 39.4
Depressive	24 16.2	21 18.3	9,1

* p < 0.05. Most patients have more than one PD-diagnosis.

Items for each diagnosis observed, 2004–2011. Absolute number and percent.

More women than men had previously been treated in compulsory care because of addiction (LVM = The Care of Alcoholics and Drug Abusers Act): 30% vs. 18% (p < .05) or in their teens 32/148 (21%) were compulsorily admitted according to The Care of Young Persons Act (LVU). Few of the patients (8%) however, had ever been admitted to psychiatric compulsive care LSPV/LPT (the Compulsory Mental Care Act). There were no gender differences.

### Retention and substance use at seven years

At seven years 94/148 (64%) of the patients in the initial cohort were retained in treatment with no illicit drug use and with present work/study (completers).

Of the 148 patients who started treatment, 117/148 (79%) were retained at 6 months, 86/148 (58%) at 12 months and 94/148 (64%) at 7 years. Women were retained in the program without interruptions to a higher degree than men (59% versus 39%), while the proportion of patients who were re-admitted after discharge was the same for both genders (35%). The average number of continuous months in treatment was the same for both sexes; 42 months (SD = 24).

Of the 84 patients who were discharged for violating the rules in the first year, 58 were re-admitted for buprenorphine treatment within the first year, while 17 were referred to methadone maintenance treatment and consequently not included in the sample. Nine patients did not seek further treatment at any other substitution program.

Alcohol problems (AUDIT) decreased significantly from baseline to follow-up (p < .001) for the whole group (see Table 4). This particularly applies to women, who initially had a higher alcohol consumption as a group than men (p < .05). All 33 patients (22%) with a high-risk consumption of alcohol, according to AUDIT and pathological blood samples indicating high alcohol consumption (B-PEth) were successfully treated with disulfiram (Antabuse) or acamprosate (Campral). From the one-year follow-up and throughout the study they were no longer heavy consumers of alcohol.

**Table 4:** **T-scores for men and women on AUDIT at three time points, 2004–2011**

Assessment	All (N = 148)	Men (N = 115)	Women (N = 33)
	M SD	M SD	M SD
Week 2	60.30 21.58	58.06 19.39	68.09* 26.81
Month 9	53.07 16.24	52.01 14.15	56.38 21.51
Month 12	50.84 15.97	49.23 13.46	54.67 20.59

* p < 0.05.

### Crime

Prior to starting treatment, men in the cohort (n = 170) had in average twice as many convictions as women; 1543 convictions (m = 11.4, SD = 9.3) versus 208 convictions (m = 5.9, SD = 5.4), t = 3.35, p >. 001).

Most of the patients in the sample (n = 148) had been sentenced to probation (73%) and more than 54% of them, males more than females, also served a prison sentence: 71 (62%) versus 9 females (27%), (Table 2). Theft and drug related crimes were the most frequent convictions.

The level of convictions in the completers group dropped from 647 for males (m = 10.6, SD = 8.0) and 112 for females (m = 3.4, SD = 3.7) at treatment start to zero at 7-years. In the dropout group, convictions decreased significantly from 896 for males at admission (m = 20.8, SD = 10.1) to 684 at follow-up (m = 11.2, SD = 3.3, t =5.92, p < .000) and from 96 convictions for females at admission (m = 8.7, SD = 7.3) to 58 at follow-up (m = 5.3, SD = 1.8), t = 1.54, ns). Throughout the study completers had significantly fewer convictions than dropouts in treatment, and lower levels continuously. In addition, women had overall fewer convictions than men.

Before the start of the treatment 17/35 of the females (49%), and 23/135 of the males (17%) had neither been convicted, imprisoned nor on probation. At the seven year follow-up, none of these patients had any convictions.

### Employment

At seven years 65/94 (69%) of the patients in the completers group were employed in regular jobs and 27 (29%) earned their living by a subsidized wage compensation (studies) while 2 (2%) conducted academic studies. Proportionally more women than men were employed or studied at the seven year follow-up (76% versus 60%). Already during the first year of treatment the clients’ work conditions changed from a precarious employment to jobs in the regular labour market, an improvement for both sexes of 30%. Employment with a public employer was the same for women as for men (12%). In addition, subsidized wage compensation increased by 19% during the first two years of follow-up. Throughout the program many patients started vocational courses or shifted to more advanced studies. Vocational training took place predominantly within the sectors of construction and catering business for both men and women. In contrast, patients who dropped out of the program lost their employment very soon after discharge and did not resume employment over the seven years.

### Premature mortality

In the cohort (n = 170) 8 persons, one woman and seven men died (0.7% annually). Five were discharged within the first month of treatment and two were early dropouts. All patients who were discharged died more than 19 months after discharge. The causes of death were analysed by forensic autopsy protocols with toxicological analyses (AN). Seven deaths were drug-related: 6/8 overdoses with opiates and 1/8 was an intoxication of mixed substances. The female patient who died was an early dropout. Only one male patient in the completers’ group died while still in treatment. The cause of death was a non-drug related fatal car accident 1/148, (0.1% annually).

### Psychological improvement

All SCL-90 symptom scales decreased significantly from clinically elevated levels above T > 70 in average at 30 days to sub-clinical levels after 12 months in the program. The global severity index (GSI) improved from T = 78 (SD = 24.00) to T = 53 (SD = 16), (p < .000). No gender differences were observed. The patients’ sense of coherence improved from a low level: SOC_**tot**_ = 120 (SD = 26) at baseline to normal levels after one year: SOC_**tot**_ =145 (SD = 24) (p < .000). At base-line, women had a lower SOC_**tot**_ (p < .01) than men, but after one year these gender differences had disappeared (see Tables 5 and 6).

**Table 5:** **T-scores of SCL-90: GSI for men and women at five time points, 2004–2011**

Assessment	All (N = 148)	Men (N = 115)	Women (N = 33)
	M SD	M SD	M SD
Month 1	80.32 23.29	81.20 25.00	77.27 15.89
Month 3	64.36 22.34	65.16 23.29	61.55 18.66
Month 6	59.58 17.84	59.97 18.01	58.39 17.58
Month 9	56.43 16.73	57.20 16.60	54.28 17.24
Month 12	53.42 16.31	53.43 16.34	53.39 16.61

**Table 6:** **Raw scores of men and women on the Sense of Coherence Scale at five time points, 2004–2011**

Assessment	All (N = 148)	Men (N = 115)	Women (N = 33)
	M SD	M SD	M SD
Month 1	118 26	121 24	108* 27
Month 3	135 26	136 24	131 30
Month 6	139 28	140 26	138 33
Month 9	145 25	145 22	146 32
Month 12	152 22	152 19	152 28

* p < 0.05.

Psychological vulnerability was assessed by the Swedish Universities Scales of Personality (SSP). All personality scales on SSP were clinically elevated at base-line (30 days). Subscales on: anxiety, stress sensitivity, bitterness and mistrust reached a clinical cut-off level: T > 62, but were reduced to a subclinical level after 12 months (see Table 7).

**Table 7:** **T-scores of SSP for men and women at admission, 2004–2011**

Subscales	All (N = 148)	Men (N = 115)	Women (N = 33)
	M SD	M SD	M SD
Somatic trait anxiety	64.49 9.56	64.17 9.67	65.58 9.23
Psychic trait anxiety	55.68 11.75	55.97 11.91	54.67 11.29
Stress susceptibility	62.51 19.54	61.89 8.24	64.70 13.04
Lack of assertiveness	49.63 6.20	50.26 3.61	47.42* 11.13
Impulsiveness	59.89 7.95	59.70 7.73	60.55 8.77
Adventure seeking	53.26 8.91	53.38 8.61	52.82 10.02
Detachment	51.97 9.14	51.68 9.07	53.00 9.42
Social desirability	46.77 10.16	47.19 9.97	45.30 10.83
Embitterment	63.32 9.19	63.30 8.91	63.36 10.28
Trait irritability	55.77 11.00	55.18 9.99	57.82 13.94
Mistrust	63.07 8.94	62.75 8.51	64.21 10.36
Verbal trait aggression	53.85 10.82	53.44 10.43	55.27 12.14
Physical trait aggression	57.16 11.04	56.50 9.53	59.42 15.14

* p < 0.05.

Dropouts had higher severity scores than completers at baseline on four scales in SSP: somatic anxiety (p < .04), stress susceptibility (p < .05), embitterment (p < .01) and mistrust (p < .08) indicating higher levels of psychological discomfort. In a previous report from the four year follow-up of the same sample the completers were compared to dropouts. Higher levels of substance abuse, conduct disorder and a low admission age at baseline predicted dropout from treatment and relapse into drug use [62].

## Discussion

Maintenance treatment for opiate dependent subjects has increased in Sweden since 1990 but, despite the increased availability of maintenance treatment overall, drug-related deaths in Sweden [8] increased to the highest number ever in 2008 since the start in 1994 of the national registration of drugs found at autopsy (Toxreg.) [28]. From 2006 to 2011 the mortality rate in Sweden increased by 59% in the methadone programs and 37% in the buprenorphine maintenance programs [63,64]. Parts of the increase reflected the increased number of patients admitted to the programs but there was also a substantial increase of the average death rate among patients in the programs, from 1.2% in 2006 to 2.7% in 2011 [63].

The practice of discharging patients who violate the rules of treatment, has been criticised for increasing the risk of intoxication, overdose and premature death [65]. However, as Romelsjö et al. [8] point out, the fear of causing premature death has paved the way for more permissive admission rules and a lax regimen to illegal drug use in some programs, which may instead have increased the mortality. It is well known that frequent relapses and side-misuse of non-prescribed drugs increase drug craving and substance use [66,67]. In addition, side-misuse in a program might stimulate relapse and dropout also among fellow-patients [6,14].

Despite the possible hazards of discharging patients from maintenance programs, the strict abstinence rules in this program did not increase the premature mortality compared to other studies [63]. In the present cohort only one patient died while in treatment (0.1% annually) and by a non-drug-related car accident. This positive finding of a very low rate of premature death raises the question whether the program systematically recruited patients with a lower severity of substance use and related problems? The answer is negative. Comparisons on several clinical and back-ground variables demonstrated that the patients had on average eight years of regular dependence on heroin and poly-drug use of the same severity as opiate dependent patients in other maintenance programs [12,21,65-67].

The monitoring of the program is, to our mind, a more important factor than patient selection. The issue of alcohol problems is vital even though that alcohol use was not an indication for discharge. High consumption of alcohol is difficult to prohibit since alcohol is a “legal substance” but alcohol nevertheless has a potential kindling effect. It sensitizes the brain and can trigger a relapse also into other drugs and narcotic substances than alcohol [67]. What is unique to this study was the intervention to reduce alcohol consumption after which most patients with a harmful consumption (22%), reduced or stopped their alcohol use. Women reduced their alcohol use to a larger extent than men. This in itself most certainly contributed to the positive outcome.

Two thirds of the patients in the sample were free from substances and problematic alcohol use at the seven year follow-up with four years continuous abstinence in average. According to the patients themselves, the consistent non-tolerance of drug use provided a strong incentive. From a biological point of view, continued opiate use destabilizes the dopamine projection regions, including amygdala and the hormonal feedback systems (HPA-axis) inducing a chronic state of stress in the patient [68,69]. Stabilization of the dopamine system may take one year of continuous abstinence to achieve, according to Koob and Volkow [68]. The dysregulation of the HPA-axis is triggered by withdrawal symptoms manifested in somatic and psychological stress of high intensity [69]. In the absence of biological markers for dopamine stabilization, significant decreases of stress susceptibility in the SSP and psychiatric symptoms (SCL-90) were indicators of this process.

Both females and males with stable abstinence improved fairly early on in treatment. This observation is in line with earlier findings [6]. A drug free treatment milieu is therefore vital for securing the optimal effect of pharmacological treatment and minimizing the hazards of intoxications and cross-tolerance development. In contrast, a permissive attitude towards excessive alcohol use and side-misuse of non-prescribed substances undermines the structure of the treatment milieu and may stimulate relapses among less motivated patients [9,14,17].

The total prevalence of personality disorders in this clinical study was high (84%) compared to the levels in other clinical samples [32], but higher than in population studies [29]. The present sample displayed the same high levels of other co-morbid psychiatric disorders and social disruptions in upbringing as most heavy opiate abusers do [14,32,41].

The criminal activity measured by convictions was reduced for the entire sample but more among the completers than among the dropouts. The convictions in the completers group dropped to zero at seven years. Women were retained in the program without interruptions to a higher degree than men (59% versus 39%), and had a much lower level of convictions over the entire study period. A Norwegian study reported a superior reduction of convictions for patients retained more than two years in maintenance programs [24]. In agreement with this and other studies, stable retention without interruptions was associated with a lower incidence of convictions [24,70]. The lower incidence of antisocial and narcissistic personality disorders in females was accordingly associated with fewer convictions [30].

The proportion of women to men was 1/3, comparable to other Swedish drug treatment programs [7,31,39]. Psychological tests at admission indicated, in agreement with other studies, higher levels of stress in females compared with males [6,30,34,41]. These and other differences in the severity of psychiatric symptoms disappeared when abstinence became stable. Women in this study seemed to benefit more from the treatment than did men despite the initially higher psychiatric symptom levels. They also had a 20% better retention than males over the entire stay. A five-year follow-up study of a gender mixed sample of substance dependent patients before methadone maintenance treatment was accessible, reported a similar superior outcome of 55% for females with two years stable abstinence compared to 30% for males [6], which emphasises the relevance of the gender issue. In the present study both women and men reported improved quality of life and self-control with concomitant reductions in drug-craving and social problems. One explanation may be that treatment with a high structure and interaction with staff like the present program appealed to women because of the enhanced relationship dimension. Women are more relationship oriented than men [71] and this facilitates attachment to their children but also to fellow employees and staff. The present treatment modality aimed to activate and nurture this re-socialisation process.

### Strengths and limitations

Since the clinical cohort is relatively small conclusions from the study have to be considered with precaution. However, the long observation time, the completeness of the data and the stability of outcome indicators from one year onwards to some extent compensated for the limited number of patients. The consecutive sampling and the systematic retrieval of data in the cohort with a high follow-up percentage give a reliable description of the group and of changes over time. Finally, the triangulation of data with interview, clinical assessment and register data supports the validity of the findings.

An important limitation is the absence of a RCT-design proper to control for confounders other than those we have analysed. A positive outcome always invites the question of self-selection. The similarities with other maintenance program samples, however, contradict the case of self-selection: the proportion of women to men (1/3), the severity of opiate use, years of injection use, the level of poly-substance abuse, the severity of psychiatric symptoms, the history of a disruptive home environment during childhood, and the high level of convictions as well as the proportion of patients with no convictions before admission provide support for the comparability of the findings.

## Conclusions

At seven years two thirds of the completers were working or studying, and maintained continuous abstinence from narcotic substances and alcohol. Criminal activities ceased. The same proportion (35%) of males and females who were discharged from the program were re-admitted and then continued treatment until the seven year follow-up. An important finding was that the strict abstinence rules in this program did not increase premature mortality in the sample.

Changes in drug use were accompanied by changes in lifestyle and criminal activities for both sexes but most for females. Despite a similar severity of drug use, more psychiatric symptoms and more psychiatric treatment at admission, females were in several aspects more successful than males in showing more continuous abstinence, higher retention and a more stable social situation and employment at follow-up. Women also had a continuously lower level of convictions than men from treatment start and onwards.

An important addition to the program was the formalized network of officials from several authorities that continuously supported the patients. The network officials kept the focus on employment, studies and the social situation and intervened when necessary. According to information from the officials themselves, the reliable information on abstinence/use of drugs from the treatment unit made them maintain their commitment.

Although this variant of a maintenance program may not be the treatment of choice for all heroin dependent patients, the present study indicates that the enhanced structure can give important benefits for a substantial proportion of the patients and may improve the outcome and foster a meaningful life-style. Women seemed to have benefited more from the utilization of this program than did men.

### Code of ethics

The study protocol was approved by the ethics committee of Lund University (Reg. 847/2004 and 43/2011).
